# Synergistic bactericidal activity of a ginsenoside-copper nano-agent against gram-positive and gram-negative biofilm bacteria

**DOI:** 10.3389/fmicb.2026.1758802

**Published:** 2026-01-29

**Authors:** Tao Tan, Weiyi Chang, Yihan Wang, Ran Cheng, Dongsheng Yang

**Affiliations:** 1 College of Life Science, Zhuhai College of Science and Technology, Zhuhai, China; 2 College of Life Science, Jilin University, Changchun, China

**Keywords:** antibacterial activity, anti-biofilm activity, Ginsenoside Re-copper nanoparticles, ROS amplification, self-assembly

## Abstract

**Background:**

Biofilm-associated infections pose a formidable challenge due to their high tolerance to conventional antibiotics. While copper-based therapies offer a promising avenue, their clinical utility is severely limited by non-specific cytotoxicity and rapid deactivation. To address this, we engineered an intelligent, redox-responsive nanoplatform composed of Ginsenoside Re (GS) and copper (Cu^2+^), termed GSR NPs.

**Methods:**

GSR NPs were synthesized through a facile self-assembly process using GS and Cu^2+^. The nanoparticles were extensively characterized using microscopy and molecular dynamics simulations. Their physicochemical stability, redox-responsiveness, reactive oxygen species (ROS) generation, and antibacterial efficacy were evaluated against *S. aureus* and *E. coli*. Additionally, biofilm disruption capabilities and *in vitro* biocompatibility were assessed.

**Results:**

Characterization indicated the formation of uniform, ultra-small nanospheres stabilized by coordination and hydrogen bonds. GSR NPs remained stable in physiological buffers but exhibited responsive behavior in reducing microenvironments, triggering the release of active components and ROS generation. Consequently, GSR NPs displayed potent antibacterial activity and effectively disrupted established biofilms of both *S. aureus* and *E. coli*, far surpassing the efficacy of individual components. Mechanistic investigations suggest a multi-pronged attack involving physical disruption, oxidative stress induction, and metabolic suppression. Furthermore, the nanoparticles demonstrated favorable biocompatibility with negligible cytotoxicity toward mammalian cells *in vitro*.

**Conclusion:**

This work presents GSR NPs as a highly efficient and potentially low-toxicity antibacterial strategy. By overcoming the limitations of free copper ions, GSR NPs offer a promising therapeutic alternative for combating challenging biofilm-related infections.

## 1 Introduction

The escalating crisis of antibiotic resistance is a defining challenge to global public health, a crisis profoundly exacerbated by bacterial biofilm formation (Ikuta et al., 2020; Ciofu et al., 2022). Pathogens such as *Pseudomonas aeruginosa*, *Staphylococcus aureus*, and *Escherichia coli* readily form these complex, matrix-encased communities during infection (Wang et al., 2025; Garnett et al., 2012). Within this protective matrix, bacteria exhibit a 10- to 1,000-fold increase in tolerance to both antibiotics and host immune responses, leading to persistent, hard-to-treat infections commonly associated with medical devices and chronic wounds (Ciofu et al., 2022). The failure of conventional antibiotics against biofilms makes the development of alternative therapeutic strategies an urgent priority.

Among these, inorganic antimicrobial agents, particularly copper, have garnered significant attention due to their potent, broad-spectrum bactericidal activity (Ge et al., 2025). Physiologically, copper is an essential trace element for humans, playing a vital role as a cofactor in numerous enzymatic processes (Tapiero and Tew, 2003). The antimicrobial efficacy of copper stems from a multi-pronged mode of action: it disrupts the bacterial membrane, leading to increased permeability; it catalyzes the generation of highly destructive reactive oxygen species (ROS) through Fenton-like reactions; and it inactivates essential metabolic enzymes, thereby collapsing cellular respiration (Liu F. et al., 2024; Huang et al., 2024). Despite this potent activity, the clinical translation of free copper ions faces significant hurdles. A critical limitation is the potential toxicity of copper to humans. At effective bactericidal concentrations, excess copper ions exhibit significant cytotoxicity toward mammalian cells and can induce systemic damage via oxidative stress accumulation, resulting in a narrow therapeutic window (Li et al., 2025). Moreover, their efficacy is compromised in biological environments where they are readily chelated and neutralized. A final critical limitation is their poor penetration into the dense extracellular polymeric substance (EPS) matrix of mature biofilms, limiting their effectiveness against the very infections they are meant to treat (He et al., 2025).

To address this therapeutic dilemma, nanotechnology provides a strategic framework for harnessing copper’s potency while mitigating its intrinsic flaws (Landa et al., 2025). By engineering copper-based nanomaterials, it is possible to harness the antimicrobial power of copper while mitigating its associated problems. Nanocarriers, such as carbon-based materials (Díez-Pascual, 2020), inorganic porous structures (Castillo and Vallet-Regí, 2021), natural polymers (Wrońska et al., 2023), and metal–organic frameworks (MOFs) (Lin et al., 2023), can serve as reservoirs for copper ions. These systems provide several key advantages: a high surface-area-to-volume ratio enhances interaction with bacteria (Xie et al., 2023); the “enhanced permeability and retention” (EPR) effect allows for passive accumulation at infection sites (Jadhav et al., 2024); and tailored surface chemistry can improve biofilm penetration (Lv et al., 2023). Specifically, constructing nano-delivery systems by chelating copper ions with organic ligands is a highly promising strategy. This approach not only stabilizes the copper ions, preventing their premature release and deactivation, but also enables sophisticated control over their therapeutic action (Rauf et al., 2024). The ligands currently employed are broadly classified into two main groups. The first group consists of organic small molecules (e.g., amino acids, citric acid, polyphenols), which confer a combination of chelation, reduction, and stabilization capabilities (Rakshit et al., 2023; Li Y. et al., 2022; Li X. et al., 2022); polyphenols are particularly noteworthy for their synergistic antibacterial properties (Liu T. et al., 2024). The second group comprises polymer ligands. Within this class, synthetic polymers such as polyvinylpyrrolidone (PVP) and polyethylene glycol (PEG) primarily leverage steric hindrance to prevent nanoparticle aggregation but are characteristically devoid of inherent biological functions (Javed et al., 2017; Guerrini et al., 2018). Conversely, natural polymers like chitosan provide steric stabilization and valuable bioactivities, including antimicrobial and biocompatible properties. However, their effects are often limited, and precisely controlling their properties remains challenging (Ghazzy et al., 2023). Therefore, there remains a pressing need for a ligand that merges the stability of engineered systems with the potent, synergistic bioactivity of compounds.

Ginsenosides, the principal bioactive constituents of *ginseng*, are a class of triterpenoid saponins (Li Y. et al., 2022; Li X. et al., 2022). These natural small-molecule compounds exhibit a diverse range of pharmacological activities, including potent anti-inflammatory and immunomodulatory effects, and are widely recognized for their exceptional biosafety profile (Ratan et al., 2021). Recently, their potential as novel antimicrobial and anti-biofilm agents has garnered significant scientific attention (Xue et al., 2020). Ginsenoside Re (GS), a representative saponin, is a promising candidate (Wang et al., 2020). Its amphiphilic molecular structure enables it to intercalate into the lipid bilayer of bacterial membranes, thereby disrupting membrane fluidity and potential, ultimately leading to cell death (Xue et al., 2020). Furthermore, studies have demonstrated that GS can effectively interfere with bacterial quorum sensing systems to inhibit the early biofilm formation stages, namely bacterial attachment and microcolony development (Wu et al., 2014). Crucially, the chemical structure of GS, rich in hydroxyl (-OH) groups, provides abundant lone-pair electrons, making it an ideal natural and biocompatible ligand for chelating metal ions such as Cu^2+^. Utilizing the inherently low toxicity and biological activity of GS as a carrier for copper represents an innovative approach to developing a next-generation antimicrobial nanomaterial with enhanced biocompatibility and safety.

**SCHEME 1:**
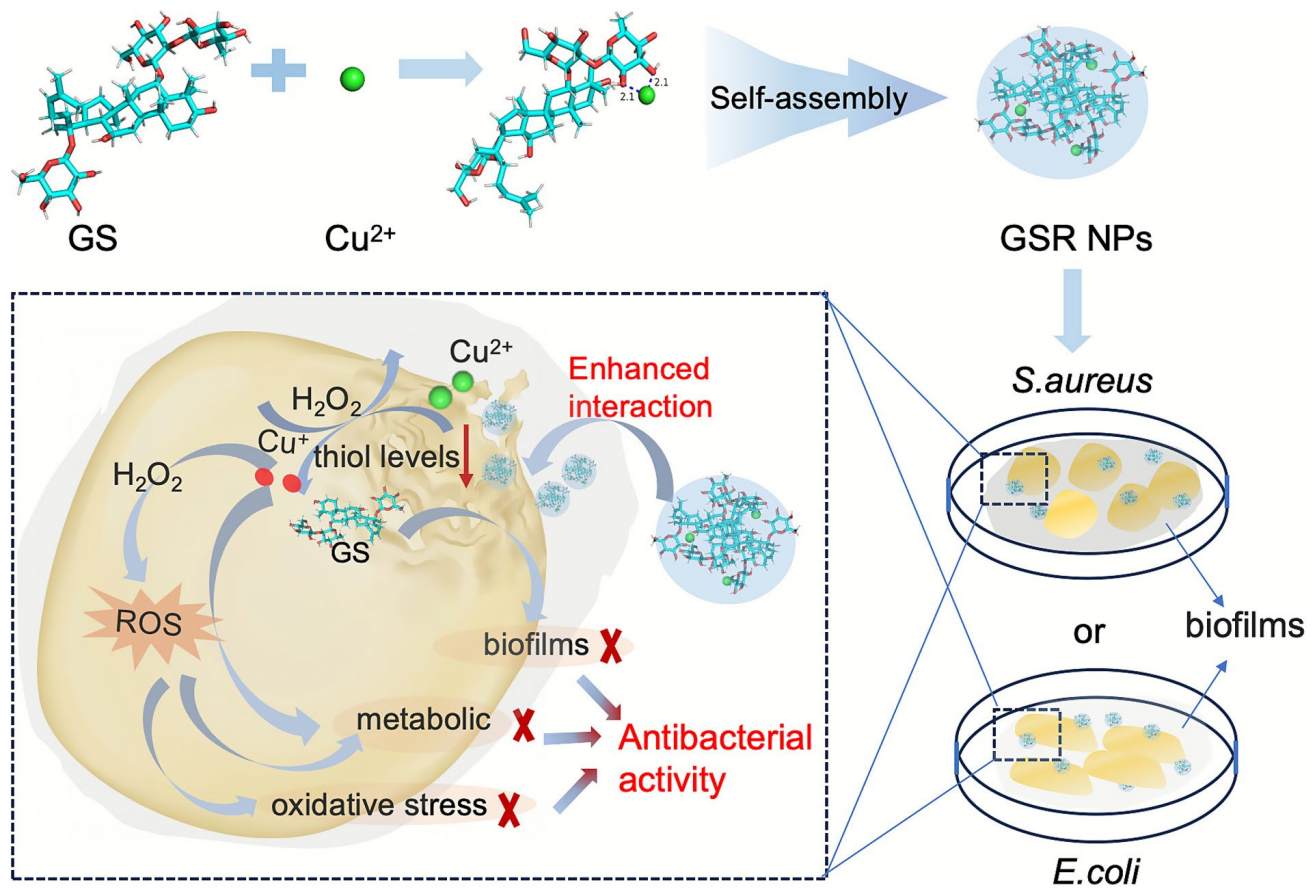
Schematic illustration of self-assembly and antibacterial mechanism of the GSR NPs.

With this in mind, we report the design and synthesis of a nanotherapeutic agent formed by the chelation of GS with Cu^2+^, which self-assembles via non-covalent intermolecular forces into discrete nanoparticles. This nano-architectural design is engineered with the aim of achieving a multi-pronged, cooperative antimicrobial effect (Scheme 1). First, the ultrasmall size of the nanoparticles is expected to facilitate their penetration into the biofilm matrix and enhance interactions with bacterial cells. Second, upon exposure to the thiol-rich microenvironment typical of bacterial infections (e.g., high glutathione concentration or equivalent reducing agents) (Zhang et al., 2023), the chelated Cu^2+^ is proposed to be reduced to the Cu^+^ species, triggering the simultaneous release of GS molecules. Finally, this localized, high-concentration release of two distinct antimicrobial agents initiates a multi-modal mechanism: copper ions are intended to induce oxidative stress (ROS), while GS molecules concurrently compromise membrane integrity. This coordinated action on multiple cellular targets is designed to produce a potent combined effect, leading to effective bacterial inhibition and the disruption of their protective biofilm, presenting a promising strategy for future applications in areas such as advanced wound dressings and anti-biofilm coatings for medical devices.

## 2 Materials and methods

### 2.1 Materials

CuCl_2_, methylene blue (MB), 5,5’-Dithiobis- (2-nitrobenzoic acid) (DTNB), and L-glutathione (GSH) were obtained from Shanghai Macklin Biochemical Co. Ltd. DCFH-DA was purchased from Shanghai Beyotime Biotechnology Co. Ltd. Ginsenoside Re (GS) and Alamar Blue reagent were supplied by Shanghai yuanye Bio-Technology Co., Ltd. SYTO9/PI Live-Dead Bacterial Double Stain Kit was obtained from Shanghai fushen biotechnology Co., Ltd. Dulbecco’s modified Eagle’s medium (DMEM) and calf bovine serum (CBS) were purchased from GIBCO (CA, United States).

### 2.2 Bacterial and cell culture

In this study, *Escherichia coli* (*E. coli,* ATCC 8739) and *Staphylococcus aureus* (*S. aureus*, ATCC 25923) from China General Microbiological Culture Collection Center were selected as model Gram-negative and Gram-positive bacteria, respectively. All bacterial strains were initially cultured on solid Luria-Bertani (LB) agar plates. A single colony of each strain was then inoculated into fresh sterile LB broth and incubated overnight at 37 °C with shaking. The bacterial cells were harvested by centrifugation at 5,000 rpm for 5 min and washed twice with phosphate-buffered saline (PBS; 10 mM, pH 7.4) to remove residual medium. Finally, the bacterial suspensions were serially diluted in sterile water, plated onto LB agar plates, and incubated for colony-forming unit (CFU) enumeration.

Mouse NIH3T3 embryonic fibroblast cells were obtained from the Shanghai Cell Bank, Chinese Academy of Sciences (CAS). The NIH3T3 cells were cultured in the DMEM supplemented with 10% CBS, 100 U/mL of penicillin, and 100 μg/mL of streptomycin at 37 °C and 5% CO_2_ in a humidified incubator.

### 2.3 Preparation of GSR NPs

To synthesize GSR NPs, 20 mg of GS was dissolved in 2 mL of a 25% (v/v) ethanol aqueous solution with stirring at 50 °C. The pH of the solution was adjusted to 8.0 using 1 M NaOH, followed by the slow dropwise addition of 0.2 mL of a 10 mg/mL CuCl_2_ solution. After a reaction time of 1.5 h, 4 mL of anhydrous ethanol was added to the mixture. The resulting suspension was centrifuged at 5,000 rpm for 5 min. The precipitate was collected and resuspended in 4 mL of anhydrous ethanol for storage, yielding the final product designated as GSR NPs.

### 2.4 Characterization of GSR NPs

GSR NPs dispersed in PBS via bath sonication, following by determining the particle size distribution and zeta potential through dynamic light scattering (DLS; Nanoplus-3, Micromeritics). Morphology was characterized using transmission electron microscopy (TEM; Tecnai G2 F20 S-Twin, FEI) after negative staining with uranyl acetate. High-resolution HAADF-STEM imaging and elemental mapping were conducted on a Tecnai G2 F30 S-TWIN microscope (FEI, United States) using molybdenum grids (230 mesh) coated with amorphous carbon films. Atomic force microscopy (AFM) was performed on a Bruker Multimode-8 instrument operating in Scanasyst mode. UV–visible absorption spectra of GS, CuCl_2_, and GSR NPs were acquired using a UV-2600 spectrophotometer (Shimadzu, Japan). X-ray diffraction (XRD) patterns of GSR NPs were collected on a Bruker D8 Advance diffractometer (Germany) over a 2θ range of 3° to 90°. X-ray photoelectron spectroscopy (XPS) analysis was performed using an ESCALAB 250Xi system (Thermo Fisher). Fourier-transform infrared (FT-IR) spectra of GS and GSR NPs were recorded on a IRPrestige-21 spectrometer (Shimadzu, Japan) in the range of 4,000–400 cm^−1^. ^1^H NMR spectra were acquired on a Bruker 600 MHz spectrometer and processed using Mestrenova software (Mestrelab Research, United States).

The content of GS and copper in the GSR NPs was measured. Free GS and copper were separated from these nanoformulations by centrifuging at 5,000 rpm for 5 min. After being dispersed in PBS, the precipitate was treated with 5 mM GSH for 4 h. The GS content in the precipitate determined using a high-performance liquid chromatography (HPLC) system (Waters Alliance, United States) equipped with an Agilent ZORBAX SB-C18 column (5 μm, 4.6 × 250 mm) under the following conditions: flow rate, 1.0 mL/min; detection wavelength, 203 nm. The mobile phase consisted of 35% acetonitrile and 65% water with 0.1% phosphoric acid. And the copper content in the precipitate was quantified via flame atomic absorption spectroscopy (AAS; AA-7000, Shimadzu, Japan) using a hollow copper cathode lamp. The measurements were performed in triplicate.

### 2.5 Molecular dynamics simulation of GSR NPs

The initial system configuration was obtained by geometrically optimizing the ligand (GS) structure at the B3LYP/6-31G(d) level using Gaussian 16. All molecular dynamics (MD) simulations were then performed with GROMACS 2023.3. The small molecule ligand was parameterized with the General AMBER Force Field (GAFF), while parameters for Cu^2+^ and their coordination interactions were adopted from a published force field model (Zhong et al., 2025). The simulation system was constructed as follows: Cu^2+^ and GS molecules were placed in a cubic box at a molar ratio of 400:100, and the remaining space was filled with a water-ethanol mixture (3:1 by volume). Water molecules were described using the TIP3P model, and Cl^−^ were added to neutralize the system. Prior to production runs, the system underwent energy minimization via the steepest descent algorithm until the maximum force fell below 100 kJ/mol/nm. This was followed by a 0.5 ns NPT equilibration to stabilize temperature and density. Production simulations were carried out under the following conditions: temperature was maintained at 298.15 K using the V-rescale thermostat, and pressure was controlled at 1 bar with the C-rescale barostat. Van der Waals interactions were treated with a 10 Å cutoff, and long-range dispersions were corrected with the EnerPres method. Electrostatic interactions were computed using the Particle-Mesh Ewald method. The production phase lasted 100 ns with a 2 fs time step, and trajectories were saved every 1 ps.

After simulation, key properties-including root-mean-square deviation (RMSD), solvent-accessible surface area (SASA), number of hydrogen bonds, and radial distribution function (RDF) were computed using GROMACS built-in tools. Representative snapshots at 0, 5, 10, 40, 70, and 100 ns were extracted with VMD, and local interaction details between Cu^2+^ and ligands were visualized using PyMOL to analyze binding modes and dynamic behavior.

### 2.6 GS and copper ions release

To evaluate the drug-release profiles, GSR NPs were incubated in either PBS (pH 7.4) or PBS containing 5 mM GSH (pH 7.4) at 37 °C for 24 h. At predetermined intervals (0, 0.5, 1, 2, 4, 8, 12, and 24 h), aliquots were withdrawn and dispersed in ethanol. The released GS and copper were separated from the nanoformulations by centrifugation at 5,000 rpm for 5 min. The accumulated amounts of GS and Cu^2+^ in the supernatants were quantified using the methods described above. All measurements were performed in triplicate.

### 2.7 Detection of Cu^+^ by neocuproine color reaction

Neocuproine, a highly specific chromogenic probe for Cu^+^, forms a bright-yellow complex that enables naked-eye detection of cuprous ions (Zhu et al., 2022). A neocuproine solution was prepared in ethanol at a concentration of 1 mg/mL. Then, 100 μL of this solution was mixed with 2 mL of GSR NPs (containing 10 μg Cu/mL). After adding 100 μL of an aqueous GSH solution (5 mM), the color change was observed, and the absorbance of the mixture was recorded.

### 2.8 XPS test

The GSR NPs were incubated in PBS and PBS containing 5 mM GSH (pH 7.4) at 37 °C for 4 h. Then, 2 mL of each reacted solution was collected and freeze-dried for subsequent X-ray photoelectron spectroscopy (XPS) analysis.

### 2.9 Generation of the ROS

The •OH generation was quantified by methylene-blue (MB) bleaching: •OH-induced degradation of MB lowers its 665 nm absorbance. The GSR NPs (containing 10 μg Cu/mL) were first incubated in PBS with 0–5 mM GSH (0, 0.01, 0.05, 0.1, 1, 5 mM), MB (10 mg/mL) and H_2_O_2_ (10 mM) were then added. After 30 min reaction in the dark, UV–vis spectra were recorded from 500 to 800 nm (10 nm steps) at room temperature. In a parallel set, GSR NPs containing 0–20 μg Cu/mL (0, 2.5, 5, 10, 20 μg/mL) were mixed with 5 mM GSH, followed by the same addition of MB and H_2_O_2_ and identical spectroscopic analysis.

### 2.10 Consumption of the GSH

To quantify GSH depletion, 0.1 mL of 5 mM GSH was added to 0.85 mL GSR NPs dispersed in PBS containing 0–20 μg Cu/mL (0, 1.5, 2.5, 5, 10, 20 μg/mL). After 30 min stirring at 25 °C, 0.05 mL DTNB (3 mg/mL) was introduced and the mixture was incubated for a further 30 min; residual GSH was determined from the 412 nm absorbance. For the time-course, 0.1 mL of 5 mM GSH was mixed with 0.85 mL GSR NPs (10 μg/mL Cu^2+^) and stirred at 25 °C. At intervals (0–60 min) 0.05 mL DTNB was added, and the remaining GSH was measured at 412 nm after 30 min reaction. Background correction was performed with GSR NPs plus DTNB in the absence of GSH.

### 2.11 Antibacterial activity assay

In this study, *E. coli* and *S. aureus* were selected as model bacterial strains. The bacteria were cultured in 50 mL tubes containing 20 mL of liquid Luria Bertani (LB) medium and incubated at 37 °C on a shaker operating at 180 rpm to exponential phase, harvested by centrifugation, washed twice with PBS, and re-suspended to 1 × 10^8^ CFU/mL. Antibacterial activities were determined by broth micro-dilution in 96-well plates. One hundred microliter of 2-fold serial dilutions of CuCl_2_, GS, a GS + CuCl_2_ physical mixture, or GSR NPs (copper-equivalent concentrations: 0–80 μg/mL) were mixed with 100 μL bacterial suspension (2 × 10^6^ CFU/mL). After 16 h at 37 °C, OD_600_ was read on a microplate reader.

To explicitly determine the Minimum Inhibitory Concentration (MIC) and Minimum Bactericidal Concentration (MBC) without optical interference from the nanoparticles, standard colony-forming unit (CFU) counting assays were performed. Fifty microliter above aliquots from wells treated with CuCl_2_, GS, a GS + CuCl_2_ physical mixture, or GSR NPs (5, 10, and 20 μg/mL copper equivalents) were spread on LB agar and incubated at 37 °C until colonies appeared. All assays were performed in triplicate. Meanwhile, 50 μL aliquots from wells treated with GSR NPs (containing 10 μg Cu/mL) for 0.5, 2, and 4 h were spread on LB agar and incubated at 37 °C until colonies appeared. All assays were performed in triplicate.

### 2.12 Assessment of bacterial membrane integrity

To evaluate the impact of the treatments on bacterial membrane integrity, a flow cytometry analysis was performed using the SYTO 9/PI Live/Dead Bacterial Viability Kit. Bacteria were first incubated with CuCl_2_, GS, their physical mixture, or GSR NPs (at equivalent concentrations of 10 μg/mL Cu or 50 μg/mL GS). Subsequently, the cells were collected via centrifugation, washed three times with ice-cold PBS, and stained with the SYTO 9 and PI dual-dye solution according to the manufacturer’s protocol. The uptake of propidium iodide (PI) by cells is indicative of compromised membrane integrity. After a final wash to remove unbound dyes, the fluorescence intensities of the bacterial population were quantified using a CytoFlex flow cytometer (Beckman, United States). All assays were performed in triplicate.

### 2.13 Sample preparation for morphological characterization of bacteria

*E. coli* and *S. aureus* cells were treated with PBS, CuCl_2_, GS, a GS + CuCl_2_ physical mixture, or GSR NPs (equivalent to 10 μg Cu/mL or 50 μg GS/mL) for 4 h. After treatment, the cells were washed three times with PBS and fixed overnight at 4 °C using 2.5% glutaraldehyde solution. Subsequently, the samples were prepared for SEM morphological observation.

### 2.14 Assessment of biofilm disruption and inhibition

The biofilm disruption capacity of the GSR NPs was evaluated against preformed *E. coli* and *S. aureus* biofilms. Biofilms were established in 96-well plates by seeding each well with a 100 μL bacterial suspension (10^6^ CFU/mL) incubating for 24 h at 37 °C. After incubation, the culture medium was aspirated, and the established biofilms were gently washed twice with sterile PBS. The biofilms were then treated for 24 h at 37 °C with 200 μL of the following solutions prepared in LB medium: CuCl_2_, GS, a GS + CuCl_2_ physical mixture, or GSR NPs (equivalent to 10 μg Cu/mL or 50 μg GS/mL). For quantification of the remaining biofilm biomass, the treatment solutions were aspirated, and the wells were washed twice with PBS. The biofilms were stained with 200 μL of 0.1% (w/v) crystal violet for 10 min. The stain was then removed, and after a final washing step, the bound dye was solubilized in 200 μL of 95% ethanol. The absorbance of the solubilized dye was quantified at 570 nm using a microplate reader (Enspire, PerkinElmer, United States).

The capacity of GSR NPs to inhibit biofilm formation by *E. coli* and *S. aureus* was quantified via a crystal violet staining assay. Two hundred microliter of a bacterial suspension (10^6^ CFU/mL) containing one of the following treatments: CuCl_2_, GS, a GS + CuCl_2_ physical mixture, or GSR NPs (equivalent to 10 μg Cu/mL or 50 μg GS/mL) was added to the wells of a 96-well plate. The plates were incubated statically at 37 °C for 24 h to facilitate biofilm development. Subsequently, the culture supernatant was aspirated, and non-adherent cells were removed by washing the wells twice with PBS. The remaining biofilms were stained with 200 μL of 0.1% (w/v) crystal violet for 10 min. The stain was then discarded, and after a final wash, the bound dye was solubilized with 200 μL of 95% ethanol. The absorbance of the resulting solution, corresponding to biofilm biomass, was measured at 570 nm.

To distinguish specific anti-biofilm activity from biofilm reduction caused by bactericidal effects, a normalization assay was performed at a sub-lethal concentration (5 μg/mL), as previously described with modifications. Briefly, bacterial suspensions (10^6^ CFU/mL) were treated with 5 μg/mL GSR NPs in 96-well plates for 24 h at 37 °C. Parallel samples were prepared to quantify: (1) total biofilm biomass using the crystal violet staining method and (2) planktonic bacterial survival using the spread plate method. The data were normalized to the untreated control (set as 100% or 1.0). The Specific Biofilm Formation (SBF) index was calculated as the ratio of normalized biofilm biomass to normalized bacterial survival. An SBF index significantly lower than 1.0 indicates specific inhibition of biofilm formation independent of cell killing.

### 2.15 Intracellular ROS quantification

Intracellular ROS levels were assessed using 2′,7′-dichlorodihydrofluorescein diacetate (DCFH-DA). Freshly cultured *E. coli* or *S. aureus* cells were washed twice with PBS, and resuspended in PBS to a density of 5 × 10^7^ CFU/mL. Bacterial suspensions were treated for 4 h at 37 °C with PBS (control), CuCl_2_, GS, a GS + CuCl_2_ physical mixture, or GSR NPs (equivalent to 10 μg Cu/mL or 50 μg GS/mL), respectively. After treatment, cells were centrifuged (8,000 rpm, 5 min), washed twice with PBS, and incubated with 20 μM DCFH-DA for 60 min at 37 °C in darkness. Flow cytometry analysis (excitation/emission: 485/525 nm) quantified green fluorescence derived from DCF formation via oxidative cleavage of DCFH-DA. All assays were performed in triplicate.

### 2.16 Detection of intracellular low-molecular-weight thiols

Intracellular LMW thiol levels were quantified using a modified Ellman’s assay protocol (Erel and Neselioglu, 2014). *E. coli* or *S. aureus* cells from a fresh culture were washed, resuspended in PBS to 5 × 10^7^ CFU/mL, and treated for 4 h at 37 °C with PBS (control), CuCl_2_, GS, a GS + CuCl_2_ physical mixture, or GSR NPs (equivalent to 10 μg Cu/mL or 50 μg GS/mL), respectively. Following treatment, the cells were pelleted (8,000 × g, 5 min), washed twice with PBS, and lysed via probe sonication on ice for 10 min. The cell debris was removed by centrifugation (12,000 × g, 10 min, 4 °C) to obtain a clear lysate. The reaction was initiated by adding 100 μL of 100 mM DTNB (in 10 mM PBS, pH 8.0) to 900 μL of the lysate. After a 30-min incubation in darkness at 37 °C, the absorbance of the resulting product, 2-nitro-5-thiobenzoate (TNB^2−^), was measured at 412 nm using a microplate reader. A corresponding mixture of PBS and the DTNB solution was used as the blank. All assays were performed in triplicate.

### 2.17 Assessment of general metabolic activity

The overall metabolic activity of the bacteria was assessed using an Alamar Blue (resazurin) reduction assay (Wang et al., 2021). Briefly, following treatment and washing as described in the ROS assay section, the bacterial pellets were resuspended in PBS to a final concentration of 5 × 10^7^ CFU/mL. In a 96-well plate, 100 μL aliquots of this suspension were mixed with 10% (v/v) Alamar Blue reagent. The plate was incubated for 60 min at 37 °C in darkness. The metabolic conversion of resazurin to the fluorescent product resorufin was quantified by measuring the absorbance at 570 nm using a microplate reader. Results were normalized to the PBS-treated control group. All assays were performed in triplicate.

### 2.18 Hemolysis assay of GSR NPs

The hemolytic activity of the GSR NPs was assessed against commercially sourced sheep erythrocytes (Yuanye, Shanghai, China). The erythrocytes were washed three times with cold PBS by centrifugation (1,500 × g, 10 min) and subsequently resuspended in PBS to obtain a 10% (v/v) stock suspension. GSR NP dispersions were prepared in PBS at concentrations of 2.5, 10, and 40 μg Cu/mL to test their activity against *S. aureus*. For the assay, 1 mL aliquots of these dispersions were incubated with 100 μL of the erythrocyte stock suspension. Parallel controls consisted of a negative control (100 μL erythrocytes in 1 mL PBS) for minimal hemolysis and a positive control (100 μL erythrocytes in 1 mL ultrapure water) for 100% hemolysis. Following a 2 h incubation at 37 °C, the samples were centrifuged (800 × g, 5 min) to pellet intact erythrocytes. The amount of released hemoglobin in the supernatant was quantified by measuring its absorbance at 540 nm. The hemolysis percentage was calculated using the following equation:

$$\text{Hemolysis rate}\;(\%)=(A_{n}-A_{0})/(A_{1}-A_{0})\times 100$$

where *A_n_*, *A*_0_, and *A*_1_ are the absorbances of the supernatants from the nanoparticle-treated samples, negative control, and positive control, respectively. All assays were performed in triplicate.

### 2.19 Cell viability

The cytotoxicity of GSR NPs against NIH3T3 fibroblasts was assessed via a standard MTT (3-(4,5-dimethylthiazol-2-yl)-2,5-diphenyltetrazolium bromide) assay. Cells were seeded into 96-well plates at a density of 1 × 10^4^ cells/well and cultured for 24 h (37 °C, 5% CO_2_) to ensure adherence. The seeding medium was then aspirated and replaced with 100 μL of fresh medium containing serial dilutions of the following treatments: GSR NPs, CuCl_2_, GS, and a physical mixture of GS & CuCl_2_. For all copper-containing groups (GSR NPs, CuCl_2_, and the mixture), the final copper concentrations ranged from 1.25 to 40 μg/mL. For all ginsenoside-containing groups (GSR NPs, GS, and the mixture), the ginsenoside concentrations were set to be 5-fold higher than the corresponding copper concentrations (i.e., 6.25–200 μg/mL), reflecting the 5:1 mass ratio. Complete medium without any added compounds served as the negative control. Prior to dilution, the GSR NP stock was dispersed in serum-free DMEM via bath sonication. Following a 24 h exposure period, 10 μL of MTT solution (5 mg/mL in PBS) was added to each well for a 4 h incubation. Afterward, the supernatant was carefully removed, and the resulting formazan crystals were solubilized in 200 μL of DMSO with 10 min of orbital shaking. The absorbance was measured at 570 nm with a microplate reader to determine cell viability. All assays were performed in triplicate.

### 2.20 Statistical analysis

All quantitative data are presented as the mean ± standard deviation (SD) from at least three independent experiments (*n* = 3), unless otherwise specified. Statistical comparisons were performed using a one-way analysis of variance (ANOVA), followed by Tukey’s *post-hoc* test for multiple pairwise comparisons. Differences were considered statistically significant when the *p*-value was less than 0.05. Significance levels are indicated in the figures as * for *p* < 0.05, ** for *p* < 0.01, *** for *p* < 0.001, and **** for *p* < 0.0001. All analyses were performed using GraphPad Prism software (Version 10.2.0, GraphPad Software, United States).

## 3 Results and discussion

### 3.1 Synthesis and physicochemical characterization of GSR NPs

The GSR NPs were synthesized via the coordination-driven self-assembly of Ginsenoside Re (GS) and Cu^2+^. After purification, the nanoparticles were stored in ethanol to prevent oxidation and ensure long-term stability. Prior to use, the stock solution was centrifuged and resuspended in PBS (pH 7.4) via ultrasonication. This preparation yielded a formulation with a GS concentration of 1.75 ± 0.11 mg/mL and a Cu^2+^ concentration of 0.35 ± 0.08 mg/mL.

**Figure 1:**
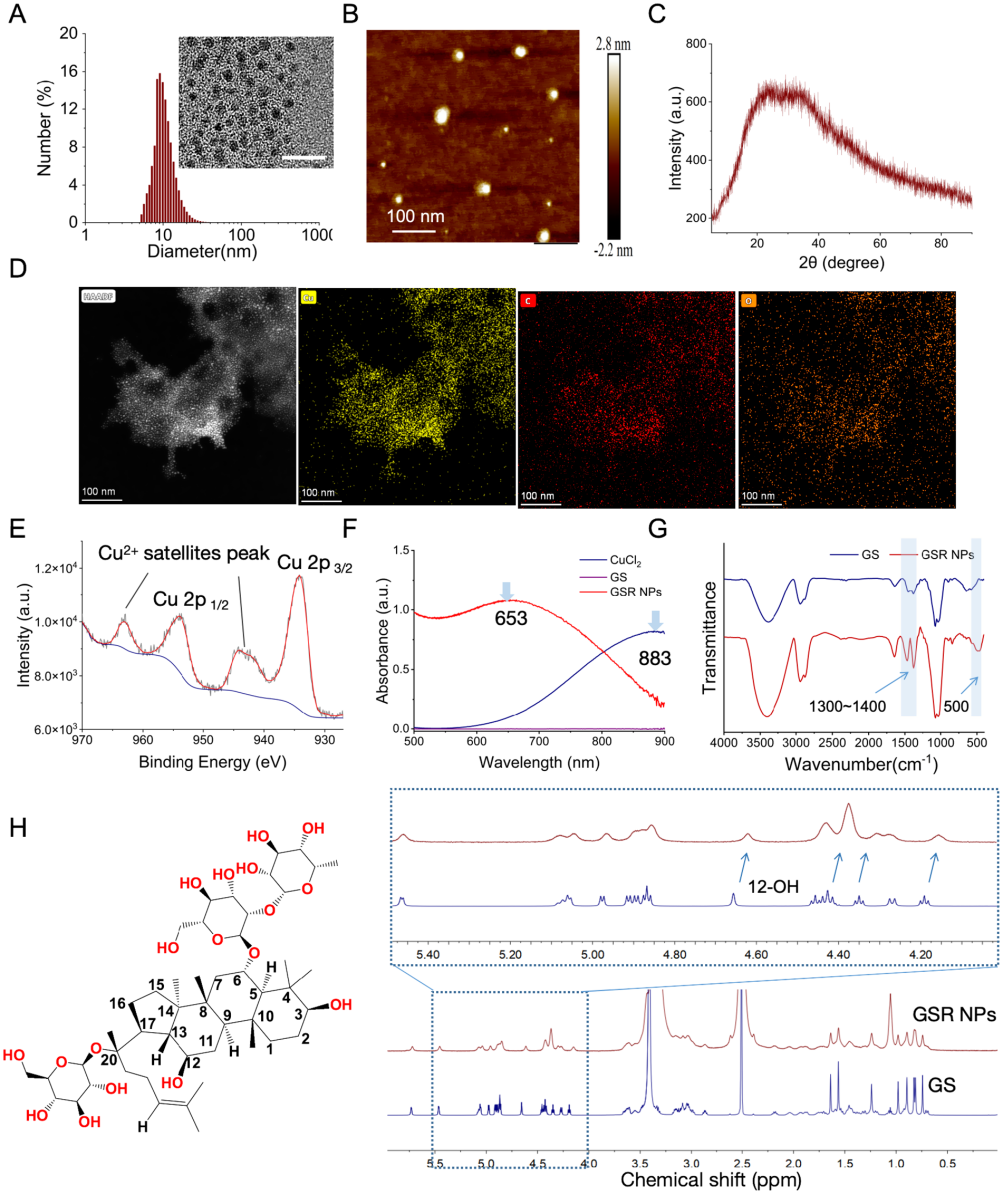
Characterization of GSR NPs. **(A)** Hydrodynamic size and TEM images GSR NPs, scale bar = 10 nm. Inset: TEM image; Graph: DLS size distribution. **(B)** AFM images GSR NPs. **(C)** XRD patterns GSR NPs. **(D)** Elemental mapping of GSR NPs. **(E)** XPS high-resolution spectra of Cu in GSR NPs. **(F)** UV–Vis spectra of CuCl_2_, GS, and GSR NPs. **(G)** FTIR spectra of GS and GSR NPs. **(H)** ^1^H-NMR spectra of GS and GSR NPs.

To understand the structural attributes of the synthesized agent, we first analyzed its morphology. TEM imaging revealed that the GSR NPs appeared as discrete, spherical nanoparticles (Figure 1A). This spherical morphology and uniform distribution were further corroborated by AFM analysis (Figure 1B). Interestingly, a comparison between the high-magnification TEM images and the HAADF-STEM elemental mapping (Figure 1D) reveals an apparent discrepancy: the mapping images show a more clustered morphology compared to the discrete particles seen in TEM. This clustering is likely an artifact of the drying process required for STEM sample preparation, where solvent evaporation concentrates the particles. Despite this drying effect, the mapping confirms the homogenous co-localization of Cu, C, and O elements, verifying the formation of a copper-ginsenoside coordination network.

The colloidal behavior of nanoparticles in physiological environments is critical for their antibacterial application. DLS analysis of the GSR NPs in PBS indicated a hydrodynamic diameter of 10.6 ± 3.3 nm with a narrow polydispersity index (PDI) of 0.28 ± 0.08. Generally, a zeta potential closer to neutral implies low electrostatic repulsion and a tendency to aggregate; here, the GSR NPs exhibited a near-neutral potential of −3.6 ± 0.9 mV. While a low zeta potential typically suggests limited electrostatic stabilization, the GSR NPs maintained colloidal integrity under our experimental conditions. Monitoring in PBS for 24 h revealed no significant aggregation, with the hydrodynamic diameter remaining consistent at 12.6 ± 4.3 nm (PDI = 0.29 ± 0.05). This stability is likely attributable to the amphiphilic nature of the surface-bound ginsenosides. We hypothesize that these bulky molecules provide steric hindrance that compensates for the weak electrostatic repulsion, thereby helping to stabilize the dispersion. Such ultra-small, stable characteristics are often considered favorable for penetrating bacterial membranes and biofilm matrices (Jiang et al., 2022). Finally, the XRD pattern exhibited a single broad diffraction halo (Figure 1C), indicating that the GSR NPs possess an amorphous structure, which is typical for metal–organic coordination polymers. Collectively, these physicochemical data confirm the successful assembly of GS and Cu^2+^ into a uniform nanostructure, providing a stable and defined platform for the subsequent antibacterial investigations.

### 3.2 Self-assembly process and characteristics of GSR NPs

The self-assembly process and coordination chemistry of the GSR NPs were elucidated using a suite of spectroscopic techniques. XPS was first employed to confirm the presence and oxidation state of copper. While the survey spectrum confirmed the elemental composition (Supplementary Figure S2), the high-resolution Cu 2*p* spectrum was definitive, displaying the characteristic binding energy peaks and strong shake-up satellite peaks that are the spectroscopic signature of Cu^2+^ (Figure 1E), aligning with standard reference data for Cu^2+^ complexes (Liu T. et al., 2024). To demonstrate that this Cu^2+^ was coordinated to ginsenoside (GS), we used UV–vis spectroscopy. As shown in Figure 1F, the broad d-d transition peak of aqueous CuCl_2_ at 883 nm was significantly blue-shifted to 653 nm upon formation of the GSR NPs, clearly indicating a change in the copper ion’s coordination environment. Similar blue shifts have been reported in other copper-hydroxyl complexes, indicative of strong ligand field splitting due to chelation (da Silva et al., 2017). FTIR spectroscopy provided direct evidence for the specific bonds involved in this coordination. The FTIR spectrum of the GSR NPs (Figure 1G) revealed a new absorption band near 500 cm^−1^, which is attributable to Cu-O stretching vibrations and absent in the spectrum of free GS. The spectral feature is consistent with the formation of metal-oxygen coordinate bonds observed in other research (Okada et al., 2014). This is further supported by the notable sharpening of bands in the 1,300–1,400 cm^−1^ region, reflecting coordination-induced conformational changes in the GS ligand.

Finally, ^1^H-NMR spectroscopy confirmed the formation of the assembly (Figure 1H). Compared to free GS, the GSR NPs exhibited global peak broadening, attributed to the combined effects of paramagnetic relaxation enhancement (PRE) from integrated Cu^2+^ and shortened T₂ relaxation characteristic of restricted molecular tumbling. Notably, the 12-OH (~4.65 ppm) and sugar ring protons (4.1–4.5 ppm) exhibited a distinct upfield shift (shielding) accompanied by significant broadening. This indicates that these hydrophilic groups are no longer exposed to the solvent but are shielded within the densely packed architecture. Collectively, these spectral features experimentally verify the transition of flexible ligands into a compact, Cu-integrated supramolecular assembly, providing direct structural evidence for the rigid confinement of ginsenoside molecules.

### 3.3 Molecular dynamics simulations reveal the assembly mechanism

**Figure 2:**
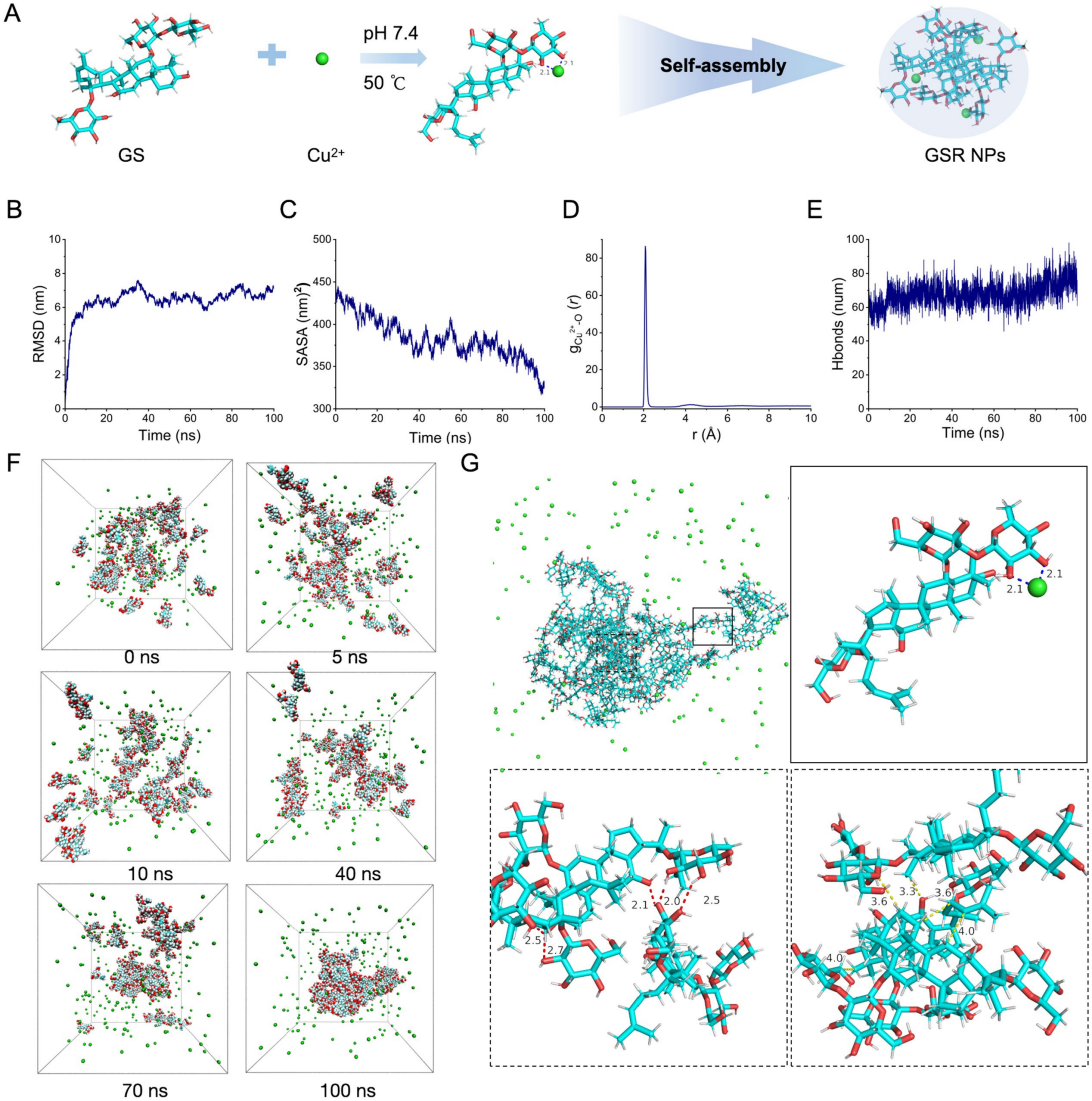
Results of molecular dynamics simulations (MDS) involving Cu^2+^ and GS. **(A)** Self-assembly process of Cu^2+^ and GS. **(B)** The time evolution curve of RMSD values for GS. **(C)** The time evolution curve of SASA values for GS. **(D)** The RDF between the hydroxyl oxygen of GS and Cu^2+^. **(E)** Dynamics of hydrogen bonding as observed in the molecular dynamics simulations. **(F)** Time-resolved snapshots of GSR NPs dynamics. Color code: Cu^2+^ (green), C (cyan), O (red), H (silver). **(G)** Detailed interaction diagram between GS or Cu^2+^. Black dashed lines denote coordination bonds, red dashed lines represent hydrogen bonds, and yellow dashed lines indicate van der Waals interactions; all distances are given in Å. Color code: Color code: Cu^2+^ (green), C (cyan), O (red), H (silver). RMSD, Root-mean-square deviation; SASA, Solvent-accessible surface area; RDF, Radial distribution function.

Complementing the spectroscopic and structural evidence presented in Figure 1, Molecular Dynamics (MD) simulations were employed to elucidate the atomic-level driving forces and conformational dynamics of the assembly (Figure 2A). First, the structural equilibrium of the system was evaluated via RMSD analysis (Figure 2B). The trajectory showed an initial increase corresponding to the reconfiguration of free ligands, followed by a plateau after ~40 ns. The stabilization of RMSD fluctuations indicates that the system reached a robust equilibrium state. Consistent with the solvent shielding effects observed in the NMR spectra, the Solvent Accessible Surface Area (SASA) significantly decreased from ~450 nm^2^ to ~325 nm^2^ (Figure 2C). This reduction confirms a structural compaction process where GS molecules progressively minimize their water exposure to form a dense core.

To pinpoint the specific interactions driving this aggregation, we analyzed the Radial Distribution Function (RDF) between Cu^2+^ and the hydroxyl oxygen of GS (Figure 2D). A sharp, intense peak was observed at 2.1 Å, which corresponds to the characteristic bond length of Cu–O coordination. This finding supports the role of Cu^2+^ as a coordination center that templates the initial assembly. Complementing this metal–ligand interaction, the total number of hydrogen bonds in the system exhibited a marked upward trend, rising from ~50 to over 70 (Figure 2E). This substantial increase suggests that intermolecular hydrogen bonds between adjacent GS ligands serve as a critical secondary force to reinforce and stabilize the supramolecular architecture.

Visual trajectory analysis (Figure 2F) further depicts this cooperative mechanism. Cu^2+^ facilitate the initial clustering, bringing ligands into proximity to trigger a cascade of non-covalent interactions. Detailed structural analysis of the final cluster (Figure 2G) highlights the synergistic nature of the assembly: Cu–O coordination (2.1 Å) acts as the primary linkage, supported by a dense network of intermolecular hydrogen bonds (2.0–2.7 Å) and van der Waals contacts (3.3–4.0 Å). Collectively, these MD results provide a molecular rationale for the experimental observations, describing a highly ordered assembly process driven by coordination and stabilized by extensive non-covalent interactions.

### 3.4 GSH-triggered nanoparticle disassembly and Fenton-like chemistry

**Figure 3:**
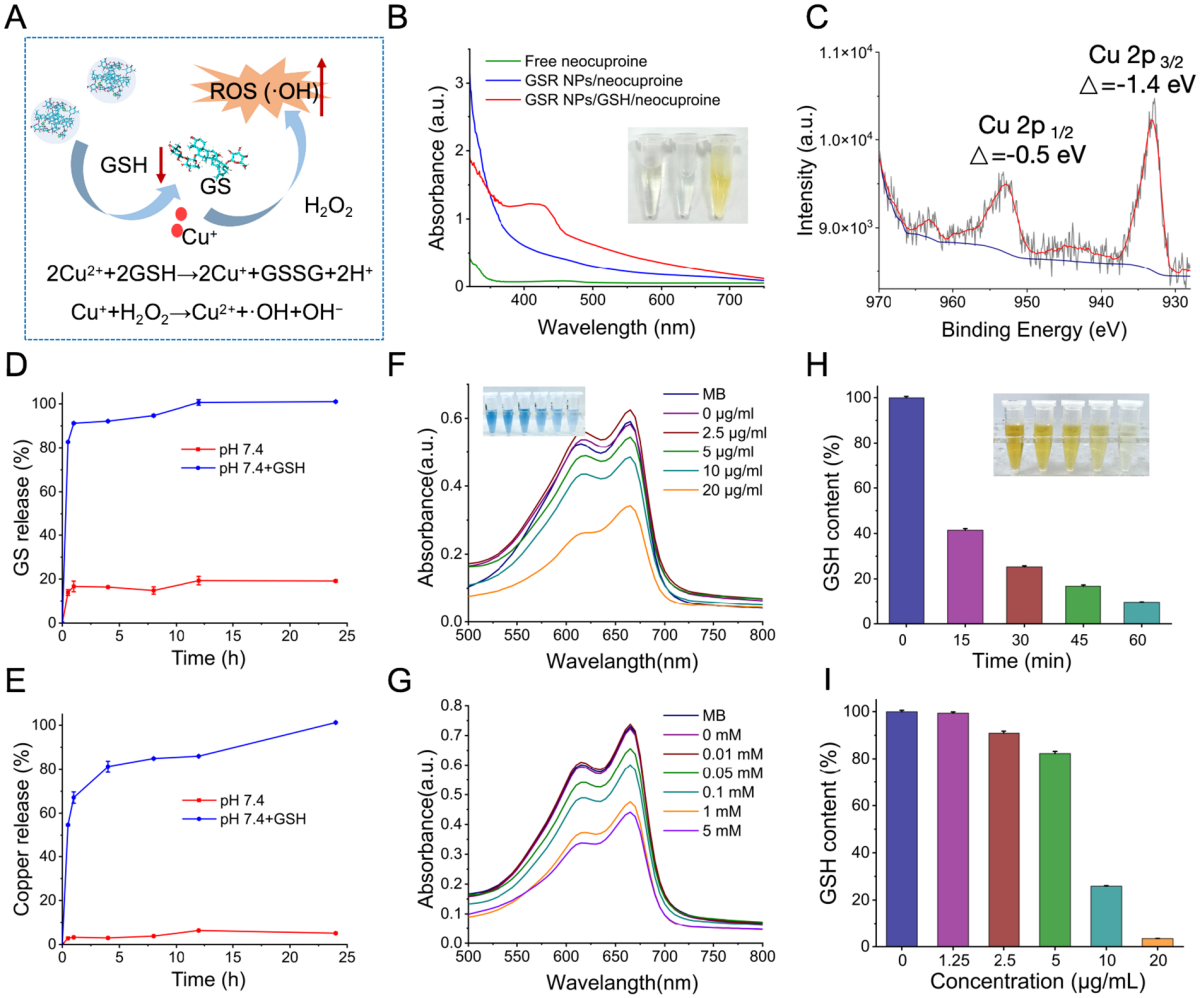
Redox-responsive drug release and catalytic ROS generation mechanism of GSR NPs. **(A)** Schematic illustration of the proposed mechanism. **(B)** UV–vis absorbance spectra demonstrating GSH-triggered Cu^+^ generation. **(C)** XPS spectrum of the Cu 2p region for GSR NPs after incubation with GSH. **(D,E)** Cumulative release profiles of **(D)** GS and **(E)** copper from GSR NPs in PBS (pH 7.4) with or without the addition of 5 mM GSH. **(F)** Concentration-dependent •OH generation (MB bleaching) by GSR NP. **(G)** GSH-enhanced ROS production: MB probe bleaching accelerates with increasing GSH. **(H)** Time-dependent depletion of GSH after incubation with GSR NPs. **(I)** Dose-dependent depletion of GSH after incubation with varying concentrations of GSR NPs. Data are presented as mean ± SD (*n* = 3).

We hypothesized that the antibacterial mechanism of GSR NPs is initiated by their disassembly upon exposure to the high levels of low-molecular-weight (LMW) thiols (such as GSH or equivalent reducing agents) characteristic of the bacterial microenvironment (Figure 3A). This strategy aligns with recent designs of tumor- or bacteria-microenvironment responsive nanomedicines (Shi et al., 2024; Kang et al., 2024). This process involves the reduction of Cu^2+^ to the Fenton-active Cu^+^ state, leading to nanoparticle dissociation and the release of its therapeutic payloads. The released Cu^+^ can then participate in Fenton-like chemistry, reacting with endogenous H_2_O_2_ to generate highly cytotoxic ROS, thereby inducing lethal oxidative stress (Chen Y. et al., 2025). To validate this hypothesis, we first sought to confirm the GSH-mediated generation of Cu^+^. Using the Cu^+^-specific chelator neocuproine, we observed the appearance of its characteristic absorbance peak at 450 nm only after GSR NPs were treated with 5 mM GSH (Figure 3B). This was accompanied by a distinct visual color change (inset), providing clear spectroscopic and visual confirmation. This valence shift from Cu^2+^ to Cu^+^ was further corroborated at the atomic level by XPS. As shown in Figure 3C, the Cu 2*p*_1/2_ and Cu 2*p*_3/2_ binding energy peak shifted to a lower value post-GSH treatment, a characteristic signature of Cu^2+^ reduction consistent with previous reports on copper-based Fenton agents (Chen Y. et al., 2025). Finally, to validate the responsive nature of the platform, we examined the drug release profiles under different conditions. In a control experiment mimicking physiological blood circulation (pH 7.4 buffer), the nanoparticles remained relatively stable, with negligible leakage of either copper ions or GS over 24 h (Figures 3D,E). This stability is crucial for minimizing systemic toxicity. However, the upon introducing 5 mM GSH to mimic the reductive potential encountered within bacterial biofilms or intracellular environments, the nanoparticles underwent rapid disassembly, triggering a burst release of both payloads. While we acknowledge that this static *in vitro* assay represents a simplified model compared to the dynamic and heterogeneous environment of an actual infection site, the data effectively demonstrates the intrinsic redox-sensitivity of the GSR NPs. This distinct difference suggests that the nanoparticles are designed to remain inert during transport but activate in reductive conditions, providing a rationale for the potent antibacterial efficacy evaluated in the subsequent sections.

### 3.5 GSR NPs mediate Fenton-like chemistry and GSH depletion

Following the release, we investigated whether the liberated copper could initiate oxidative stress. We employed MB degradation assay as a preliminary chemical indicator to assess the potential for hydroxyl radical (•OH) generation, as •OH attacks MB, reducing its absorbance at 665 nm (Liu T. et al., 2024). To address the non-specific nature of this assay, our experimental design incorporated strict internal controls. As shown in Figure 3F, in the absence of GSR NPs (0 μg/mL trace), the absorbance of MB remained stable even in the presence of H_2_O_2_ and GSH, indicating that GSH alone does not induce significant MB degradation under these conditions. Similarly, Figure 3G (0 mM GSH trace) shows that without GSH, GSR NPs alone induced minimal MB degradation, suggesting that the reduction of Cu^2+^ to Cu^+^ by GSH is a prerequisite for the observed activity. However, when all components (GSR NPs, GSH, and H_2_O_2_) were present, a concentration-dependent decrease in MB absorbance was observed. These results are consistent with copper-mediated redox activity, where reducing agents convert chelated Cu^2+^ to Cu^+^, potentially facilitating the generation of ROS via Fenton-like processes.

To further corroborate the redox capability, we monitored the consumption of GSH using Ellman’s assay. Since GSH acts as the reductant in this process, its depletion serves as a direct marker of the redox reaction. As shown in Figures 3H,I, incubation with GSR NPs led to a rapid reduction in free GSH levels in both a time- and concentration-dependent manner. This substantial depletion indicates that bacterial antioxidant defenses (such as GSH) are actively consumed in the reduction of copper. Similar GSH-depletion mechanisms have been identified as a key factor in overcoming bacterial antioxidant defenses in other metal–organic frameworks (Chen Y. et al., 2025).

Collectively, these chemical investigations suggest a dual mode of biochemical attack: the GSR NPs not only generate cytotoxic ROS but simultaneously deplete bacterial antioxidant capacity, thereby creating an oxidative imbalance favorable for bacterial killing.

### 3.6 Antibacterial effect and MIC/MBC determination

**Figure 4:**
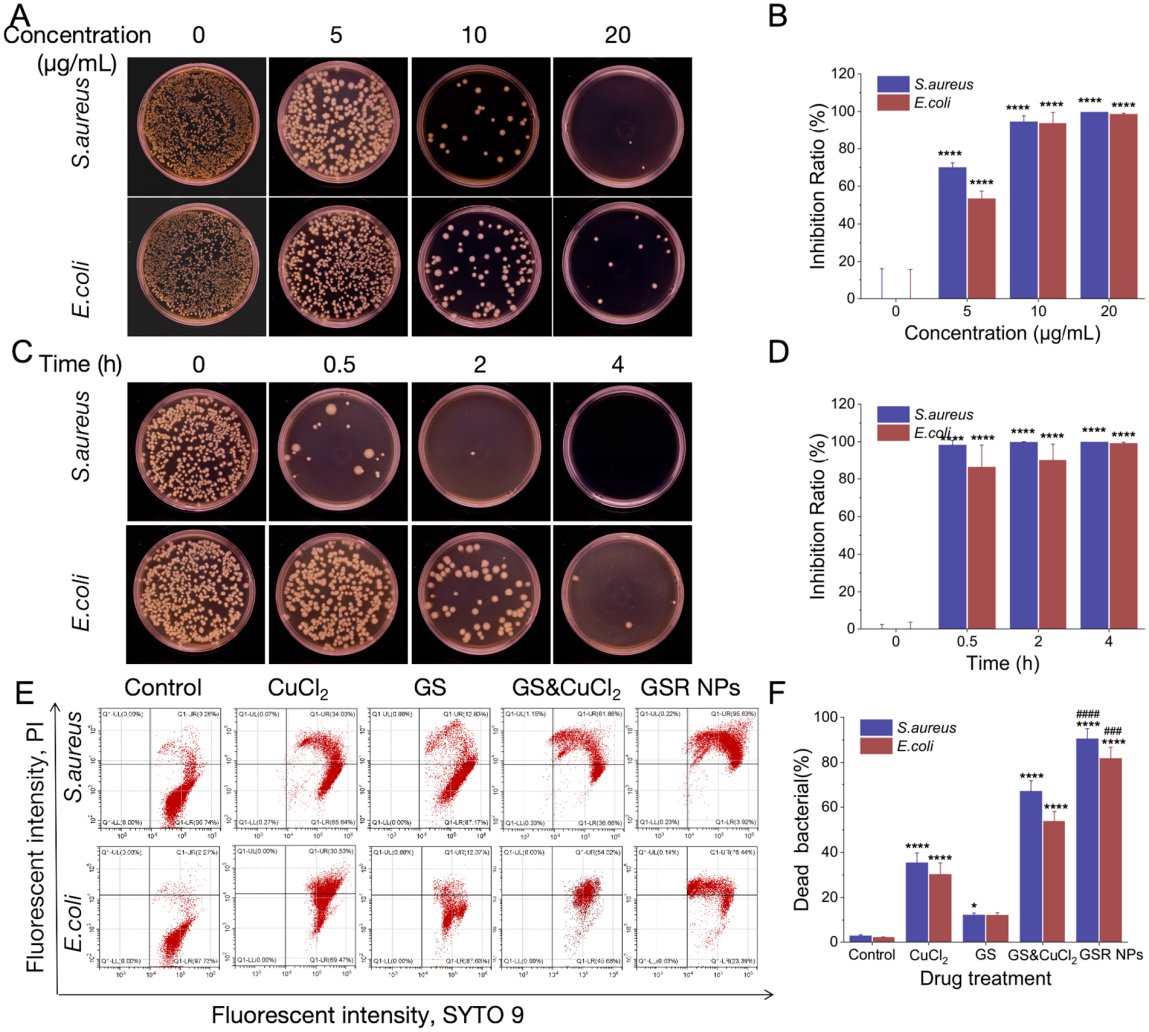
*In vitro* antibacterial activity and mechanism of GSR NPs against *S. aureus* and *E. coli*. **(A)** Representative images of agar plates showing the inhibition of bacterial colony formation for *S. aureus* and *E. coli* after treatment with GSR NPs at various concentrations. **(B)** Quantitative analysis of the inhibition ratio corresponding to the plate count assay in **(A)**. **(C)** Representative images of agar plates showing the time-course killing effect. Bacteria were treated with GSR NPs (10 μg/mL) for different durations. **(D)** Quantitative analysis of the inhibition ratio from the time-kill assay in **(C)**. **(E)** Flow cytometry analysis of bacterial membrane integrity using SYTO 9 (live cell stain) and PI (dead cell stain). **(F)** Quantification of the percentage of dead bacteria from the flow cytometry data presented in **(E)**. Data are presented as mean ± SD (*n* = 3). Statistical significance is indicated by *p < 0.05, **p < 0.01, ***p < 0.001, and ****p < 0.0001 compared to the ontrol group; ^###^*p* < 0.001 and ^####^*p* < 0.0001 compared to the GS&CuCl_2_ physical mixture group.

To accurately quantify the antibacterial efficacy, we employed the standard colony-forming unit (CFU) counting method. While initial optical density (OD600) measurements provided a preliminary assessment (Supplementary Figures S3, S4), the intrinsic turbidity of the nanoparticle suspensions interfered with absorbance readings, necessitating the use of CFU plating as the definitive method for determining Minimum Inhibitory Concentration (MIC) and Minimum Bactericidal Concentration (MBC). First, we assessed the concentration-dependent efficacy. Visual inspection of the agar plates revealed a dose-dependent decrease in CFUs for all formulations (Figure 4A; Supplementary Figure S5). Quantitative analysis confirmed these observations and highlighted the enhanced performance of GSR NPs. At a concentration of 10 μg Cu/mL, GSR NPs significantly inhibited bacterial growth, reducing *S. aureus* and *E. coli* colonies by over 90% compared to the control. Consequently, the MIC was determined to be 10 μg/mL. Furthermore, at 20 μg/mL, no viable colonies were observed, indicating reduction to below the limit of detection (>99.9% killing efficiency). Thus, the MBC was determined to be 20 μg/mL (Figure 4B).

In contrast, the control groups (free CuCl_2_, GS, and their physical mixture) failed to achieve complete inhibition within the tested concentration range (Supplementary Figures S3–S5). Even at high concentrations, significant bacterial survival was observed, indicating that their MIC and MBC values are >80 μg/mL. This substantial difference validates the synergistic design of the GSR NPs, where the co-delivery amplifies the antibacterial potency beyond that of the individual components.

Next, to investigate the bactericidal kinetics, a time-kill assay was conducted using GSR NPs at the MIC level (10 μg/mL). The results demonstrated a rapid killing action, with a marked reduction in visible colonies observed after just 2 h of incubation, leading to near-total reduction by 4 h (Figure 4C). The corresponding quantitative data confirmed this potent, time-dependent bactericidal profile (Figure 4D). Comparison with the 16 h data suggests that while 10 μg/mL induces rapid bacterial death initially, a higher concentration (MBC, 20 μg/mL) is required to prevent potential regrowth over extended periods. Collectively, these findings establish that GSR NPs exhibit potent bactericidal activity that is both concentration-dependent and time-dependent.

### 3.7 GSR NPs exert potent bactericidal activity by disrupting bacterial membrane integrity

To elucidate the bactericidal mechanism of GSR NPs, we quantified their impact on bacterial membrane integrity using a flow cytometry-based SYTO 9/PI dual-staining assay. This method distinguishes between cells with intact membranes (SYTO 9-positive, live) and those with compromised membranes (PI-positive, dead) (Yu et al., 2025). A clear hierarchy in membrane-disrupting efficacy was observed (Figures 4E,F). As anticipated, GS alone exhibited a negligible effect. In contrast, CuCl_2_ induced moderate membrane damage, rendering approximately 30% of the bacterial population PI-positive (*p* < 0.0001 vs. Control). Notably, the physical mixture of GS and CuCl_2_ led to a significantly higher proportion of dead cells (>50%), suggesting a synergistic interaction where GS potentiates the membrane-damaging activity of copper. This observation aligns with recent findings that saponins can increase membrane permeability (Kundu et al., 2025), thereby facilitating the influx of metal ions. Most significantly, the GSR NPs achieved the most profound bactericidal effect. Statistical analysis confirmed that the membrane-disrupting capability of GSR NPs was significantly superior to that of the physical mixture (GS & CuCl_2_) (*p* < 0.001, indicated by “#”), with over 80% of *E. coli* and 90% of *S. aureus* becoming PI-positive after a 4 h treatment. These findings align with our plate-counting results, indicating that rapid membrane compromise is a key contributor to the GSR NPs’ antibacterial potency.

### 3.8 Visual confirmation and dual mechanism of anti-biofilm activity

**Figure 5:**
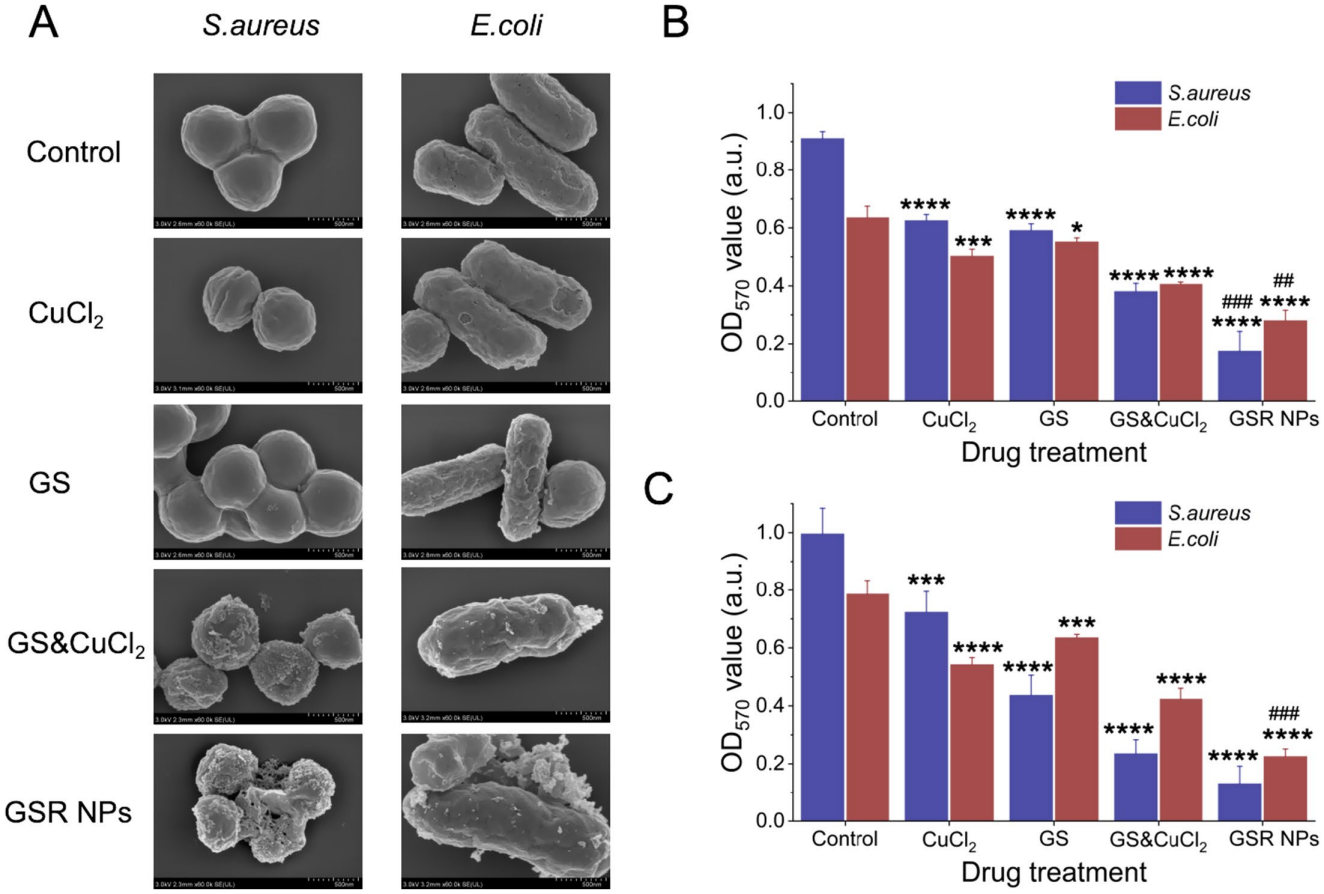
Synergistic anti-biofilm activity of GSR NPs against *S. aureus* and *E. coli*. **(A)** SEM images showing the morphological changes of *S. aureus* and *E. coli* after a 4 h incubation with CuCl_2_, GS, a physical mixture of GS & CuCl_2_, and GSR NPs. **(B)** Quantitative assessment of biofilm eradication. **(C)** Quantitative assessment of biofilm inhibition. Data are presented as mean ± SD (*n* = 3). Statistical significance is indicated by *p < 0.05, **p < 0.01, ***p < 0.001, and ****p < 0.0001 compared to the Control group; ^##^*p* < 0.01 and ^###^*p* < 0.001 compared to the GS&CuCl_2_ physical mixture group.

The extensive membrane damage quantified by our flow cytometry analysis suggests that GSR NPs exert their bactericidal effect primarily through direct physical contact with the cell envelope. This observation is consistent with the known affinity of GS for bacterial membrane proteins (Ratan et al., 2021). To provide direct, visual confirmation of this physical disruption, we employed SEM to examine the ultrastructural changes of bacteria following treatment. As shown in Figure 5A, control *E. coli* and *S. aureus* displayed smooth, intact membranes. In stark contrast, GSR NP-treated bacteria exhibited severe morphological damage, including widespread membrane wrinkling and rupture. This effect followed a clear hierarchical pattern: GS or CuCl_2_ alone caused little damage, their physical mixture induced moderate synergistic damage, but the GSR NPs were substantially more destructive, suggesting that the nanoparticle structure likely enhances this disruptive capability.

Consistent with the SEM observations, GSR NPs demonstrated potent efficacy against both pre-formed biofilms (Figure 5B) and biofilm formation (Figure 5C). While individual components (GS or CuCl2) showed statistically significant but limited reductions (*p* < 0.05, indicated by “*”), and the physical mixture offered moderate improvement, the GSR NPs were substantially more effective. Crucially, statistical analysis confirmed that GSR NPs were significantly superior to the physical mixture in both assays (*p* < 0.01 or *p* < 0.001, indicated by “#”), reducing biofilm biomass by approximately 50–80%. These results underscore the nanostructure’s distinct advantage in both preventing bacterial adhesion and eradicating established biofilms more effectively than the simple physical mixture.

To further elucidate whether this substantial reduction in biofilm formation (Figure 5C) resulted solely from bacterial killing or also involved specific anti-biofilm mechanisms, we conducted a normalization assay at a sub-inhibitory concentration (5 μg/mL) (Supplementary Figure S6). Even after normalizing for bacterial survival, the Specific Biofilm Formation (SBF) index remained significantly lower than the control (SBF < 1.0). This indicates a dual mode of action: while the potent bactericidal effect drives the massive biofilm reduction observed at higher concentrations, GSR NPs also possess the capacity to specifically inhibit biofilm formation in surviving bacteria.

### 3.9 Synergistic ROS generation and thiol depletion of GSR NPs

Having established the potent bactericidal and anti-biofilm efficacy of GSR NPs, we next sought to elucidate the underlying biochemical mechanism of cytotoxicity. We hypothesized that GSR NPs execute a multifaceted strategy centered on inducing substantial intracellular oxidative stress. To validate this, we quantified three key parameters following treatment:intracellular ROS levels, depletion of LMW thiols, and metabolic activity.

**Figure 6:**
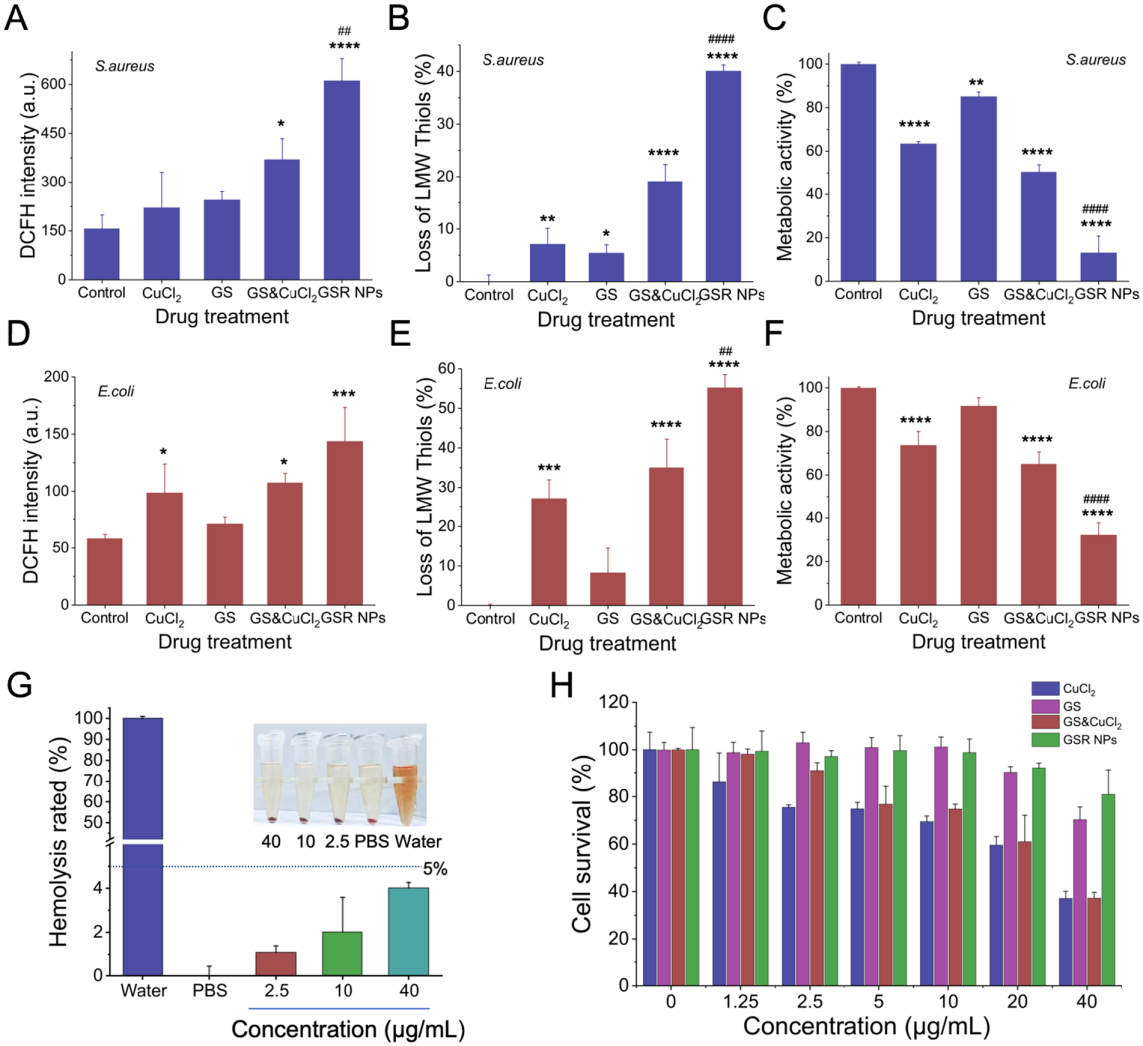
Investigation of the antibacterial mechanism and *in vitro* biocompatibility of GSR NPs. **(A,D)** Measurement of intracellular ROS levels in **(A)** *S. aureus* and **(D)** *E. coli* after treatment with various formulations, as detected by the fluorescence intensity of DCFH. **(B,E)** Quantification of the depletion of intracellular LMW thiols in **(B)** *S. aureus* and **(E)** *E. coli* following different treatments. **(C,F)** Assessment of bacterial metabolic activity of **(C)** *S. aureus* and **(F)** *E. coli* after exposure to the treatment groups. **(G)** Hemolysis assay of GSR NPs. Deionized water and PBS served as the positive and negative controls, respectively. **(H)** Cell viability of NIH3T3 cells with different treatment. Data are presented as mean ± SD (*n* = 3). Statistical significance is indicated by ^*^p < 0.05, ^**^p < 0.01, ^***^p < 0.001, and ^****^p < 0.0001 compared to the control group; ^##^*p* < 0.01 and ^####^*p* < 0.0001 compared to the cGS&CuCl_2_ physical mixture group.

First, we measured the generation of intracellular ROS in both *S. aureus* and *E. coli*. As shown in Figures 6A,D, the GSR NPs induced a significant surge in fluorescence intensity increasing by approximately 4-fold in *S. aureus* and 3-fold in *E. coli* compared to untreated controls (*p* < 0.001 or *p* < 0.0001, indicated by “*”). While the physical mixture (GS&CuCl_2_) produced a synergistic increase in ROS compared to single components, statistical analysis confirmed that the ROS burst triggered by GSR NPs was significantly more potent than even this synergistic mixture (*p* < 0.01, indicated by “##”). This highlights a distinct ‘nanoparticle advantage,’ consistent with phenomena observed in other nano-Fenton systems (Liu T. et al., 2024). Although the statistical difference in *E. coli* was less pronounced (likely due to the Gram-negative outer membrane narrowing the efficacy gap), the overall trend confirms the enhanced ROS generation capability of GSR NPs, driven by enhanced bacterium-nanoparticle interaction and localized copper concentration.

Next, we investigated the impact of this ROS surge on the bacterial antioxidant defense system by measuring the loss of LMW thiols. As hypothesized, the thiol depletion data precisely mirrored the ROS generation results (Figures 6B,E). GSR NPs caused a substantial depletion of the cellular thiol pool (approx. 40–50% loss), demonstrating that the oxidative stress significantly exceeded the bacteria’s detoxification capacity. Crucially, the effect of GSR NPs was significantly superior to that of the physical mixture (*p* < 0.01 or *p* < 0.0001, indicated by “#”). This dual action—simultaneously generating high levels of ROS while dismantling the cell’s antioxidant shield—culminated in a precipitous drop in metabolic activity (Figures 6C,F). This strategy of disabling the antioxidant defense system to amplify oxidative stress has been recently identified as a highly potent antibacterial strategy (Chen S. et al., 2025). Following treatment with GSR NPs, the metabolic activity of *S. aureus* plummeted to less than 20%, and that of *E. coli* to approximately 30% (*p* < 0.0001 vs. Control). Notably, the metabolic inhibition by GSR NPs was significantly greater than that of the physical mixture group (*p* < 0.0001, indicated by “####”), corroborating the potent bactericidal effects observed previously.

In conclusion, based on the collective evidence from the release, ROS, and membrane integrity assays, we propose a synergistic mechanism of action: The ultrasmall GSR NPs likely accumulate on the bacterial surface and penetrate the biofilm matrix. This close interaction creates a high local concentration of copper. The initial release of copper ions or surface-generated ROS induces membrane permeabilization. Consequently, the breakdown of membrane integrity facilitates the rapid influx of copper ions and the leakage of intracellular components. Upon exposure to the bacterial thiol-rich environment (either via influx or leakage), the reduction-responsive mechanism is presumed to be triggered, leading to simultaneous GSH depletion and a burst of ROS generation, causing metabolic collapse and cell death.

### 3.10 *In vitro* biocompatibility of GSR NPs

A critical prerequisite for any viable therapeutic agent is selective toxicity, meaning it must be highly effective against its target pathogen while exhibiting minimal toxicity toward host cells. To evaluate the clinical potential of GSR NPs, we conducted *in vitro* biocompatibility assessments, including *in vitro* cytotoxicity against mammalian cells and a hemocompatibility analysis.

First, we evaluated the hemocompatibility of GSR NPs, a critical parameter for any material with potential for systemic administration. A hemolysis assay was performed by incubating red blood cells (RBCs) with GSR NPs at concentrations corresponding to their bactericidal levels. As shown in Figure 6G, GSR NPs displayed favorable hemocompatibility *in vitro*. It was observed that even at the highest concentration of 40 μg/mL, the nanoparticles induced less than 5% hemolysis, a level comparable to the negative control (PBS). In contrast, the positive control (deionized water) caused complete hemolysis of the red blood cells. The inset photograph provides clear visual corroboration: the supernatants of the GSR NP-treated samples are clear, akin to the PBS control, indicating intact RBCs, whereas the water-treated sample is bright red from released hemoglobin.

Next, we assessed the cytotoxicity of GSR NPs and control formulations against a standard mammalian fibroblast cell line (NIH3T3) using a cell viability assay. As shown in Figure 6H, the GSR NPs demonstrated high cytocompatibility. Even at concentrations as high as 40 μg Cu/mL—4 fold the concentration required for potent bactericidal activity—the cell survival rate remained near 100%, indicating negligible cytotoxicity. The GS component was similarly non-toxic. In contrast, free CuCl_2_ exhibited significant, dose-dependent toxicity, with cell viability dropping to below 40% at the 40 μg/mL concentration. The physical mixture of GS&CuCl_2_ also showed comparable toxicity to CuCl_2_ alone. This crucial comparison reveals that while free copper is inherently toxic to mammalian cells, the nanoparticle formulation effectively sequesters the copper, significantly mitigating its cytotoxic effects in this cell model.

In summary, these preliminary biocompatibility studies reveal the distinct advantage of the GSR NPs. While demonstrating potent efficacy against both Gram-positive and Gram-negative bacteria, GSR NPs are well-tolerated by mammalian cells and non-hemolytic at therapeutically relevant concentrations under the tested conditions. This high degree of selective toxicity supports the potential of GSR NPs for future applications. However, it is important to note that these results are based on a single cell line and *in vitro* models. Further comprehensive *in vivo* toxicity assessments are necessary to fully validate their safety profile for clinical use.

## 4 Conclusion

In summary, this study developed a coordinate-assembled nano-agent, GSR NPs, and evaluated its potential as an antibacterial strategy. Our results indicate that the nanoparticle formulation exhibits enhanced efficacy compared to its individual components or their physical mixture. The experimental data support a multi-factorial mechanism of action: GSR NPs appear to induce significant physical damage to the bacterial membrane, which correlates with the observed reduction in biofilm biomass. Concurrently, biochemical assays suggest that the particles promote ROS generation and deplete protective thiol levels, leading to reduced metabolic activity and bacterial cell death. Importantly, this antibacterial and anti-biofilm activity is paired with favorable preliminary biocompatibility, as the nanoparticles showed low toxicity to mammalian cells and erythrocytes at effective concentrations.

It should be noted, however, that the current study relies on laboratory strains (*S. aureus* and *E. coli*) and *in vitro* models. While quantitative measurements of intracellular uptake were not performed, the observed membrane disruption and biofilm reduction strongly suggest effective bacterial interaction. The complexity of clinical infections involves dynamic host-pathogen interactions and polymicrobial environments that simple static models cannot fully replicate. Future studies will focus on evaluating the long-term stability, efficacy against clinical multi-drug resistant isolates, and performance in *in vivo* infection models to further validate the translational potential of GSR NPs for applications such as wound dressings or medical device coatings.

## Data Availability

The original contributions presented in the study are included in the article/Supplementary material, further inquiries can be directed to the corresponding author.
