# Supplementary material for: Synergistic bactericidal activity of a ginsenoside-copper nano-agent against gram-positive and gram-negative biofilm bacteria
Source: Front Microbiol. 2026 Jan 29;17:1758802. doi: 10.3389/fmicb.2026.1758802 (PMC12894357; doi:10.3389/fmicb.2026.1758802)
Supplement: Supplementary file 1 [file Data_Sheet_1.docx]

# Self-Assembled Ginsenoside Re-Copper Nanoparticles for Synergistic Antibacterial Action via ROS Amplification and Membrane Disruption

**
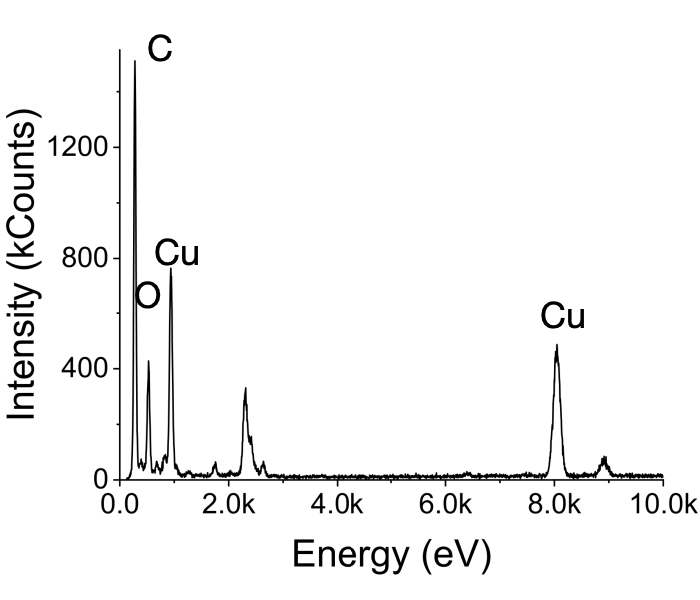
**

**Figure S1** EDS spectrum of GSR NPs.

**
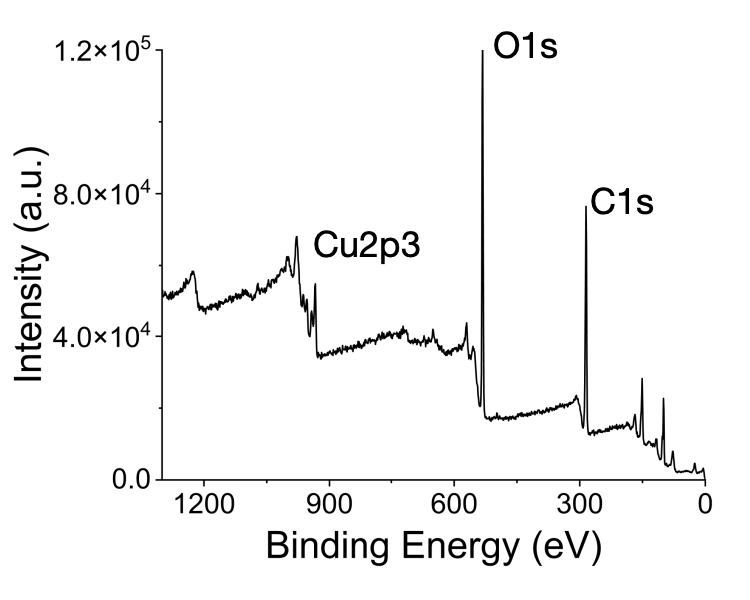
**

**Figure S2** XPS spectrum of GSR NPs.


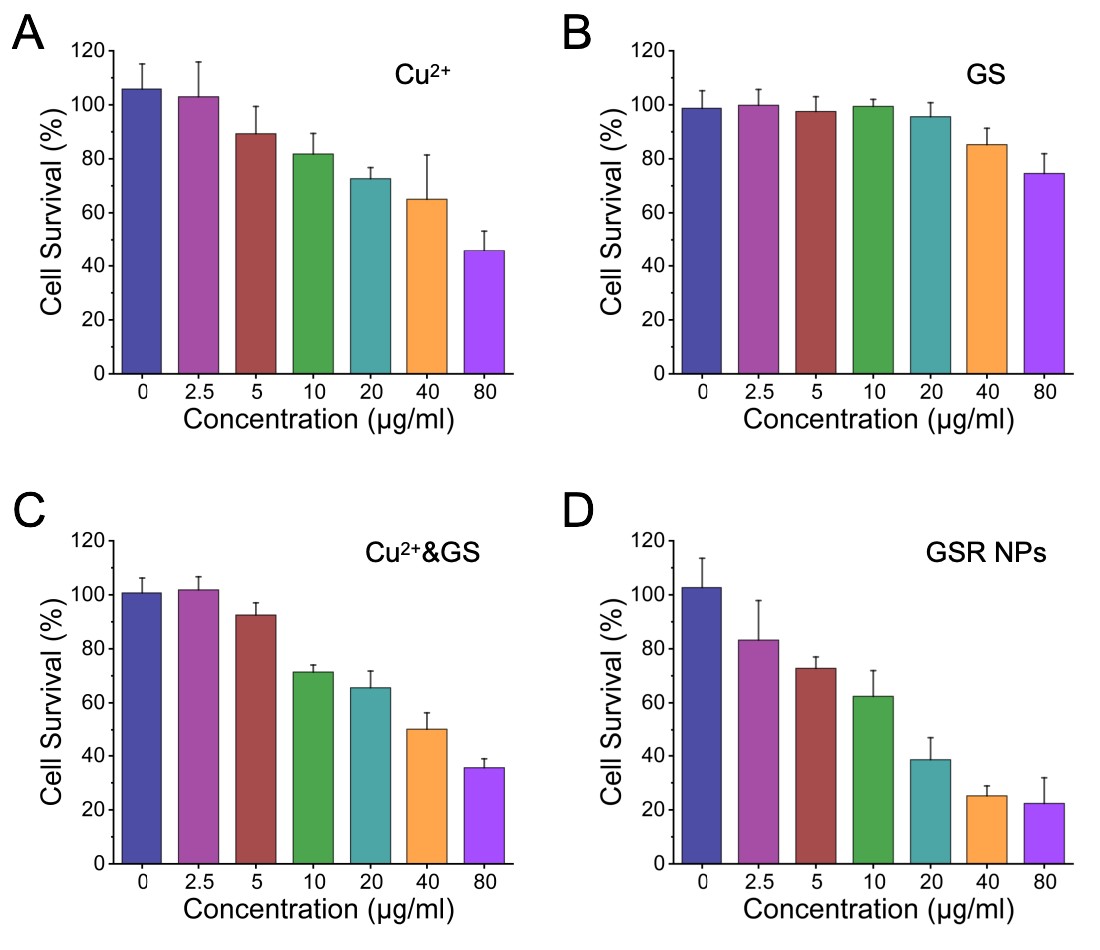


**Figure S3** Dose-dependent antibacterial activity of various formulations against *S. aureus*: (A) CuCl2, (B) free GS, (C) a physical mixture of GS & CuCl_2_, and (D) GSR NPs. Note that for GSR NPs, the apparent cell survival at high concentrations (>10 μg/mL) is overestimated due to the intrinsic turbidity of the nanoparticle suspension. Complete eradication was confirmed via CFU plating (see Figure 4).


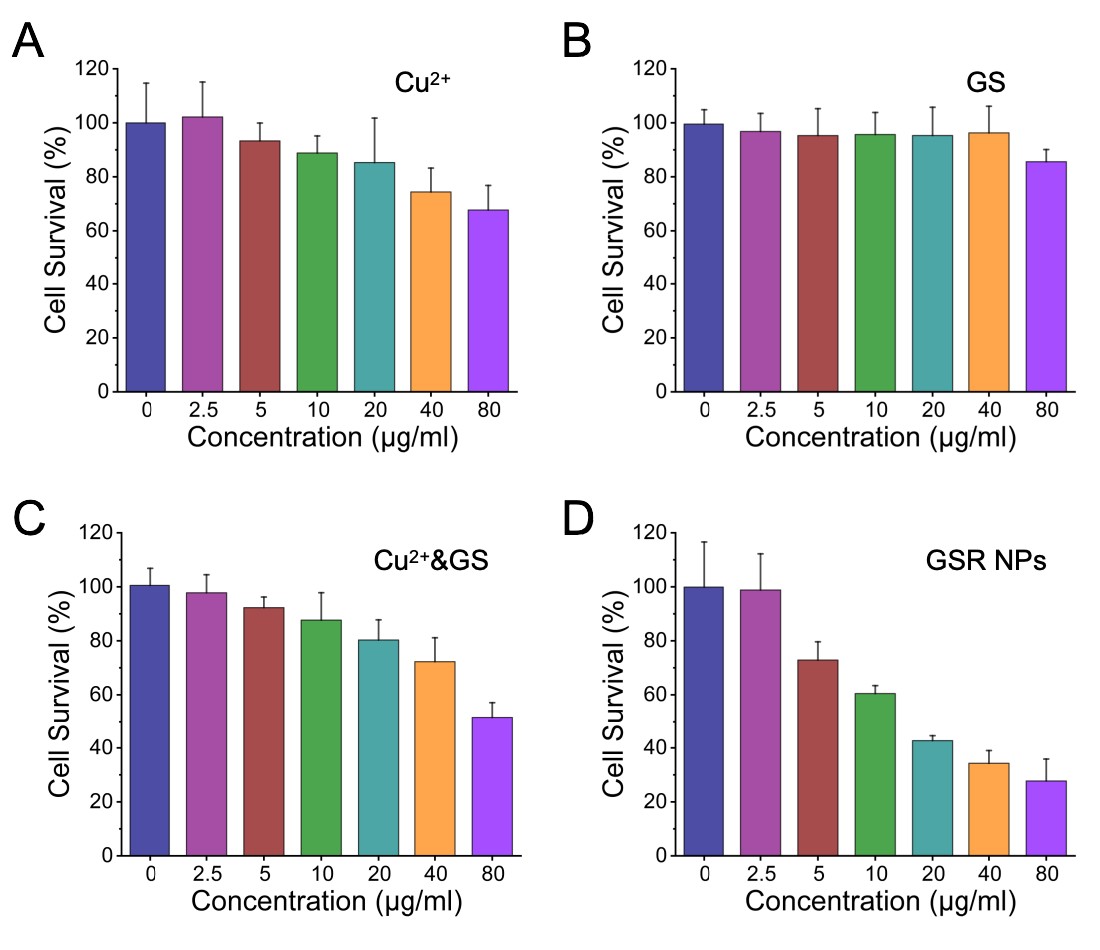


**Figure S4** Dose-dependent antibacterial activity of various formulations against *E. Coli*: (A) CuCl_2_, (B) free GS, (C) a physical mixture of GS & CuCl_2_, and (D) GSR NPs. Note that for GSR NPs, the apparent cell survival at high concentrations (>10 μg/mL) is overestimated due to the intrinsic turbidity of the nanoparticle suspension. Complete eradication was confirmed via CFU plating (see Figure 4).


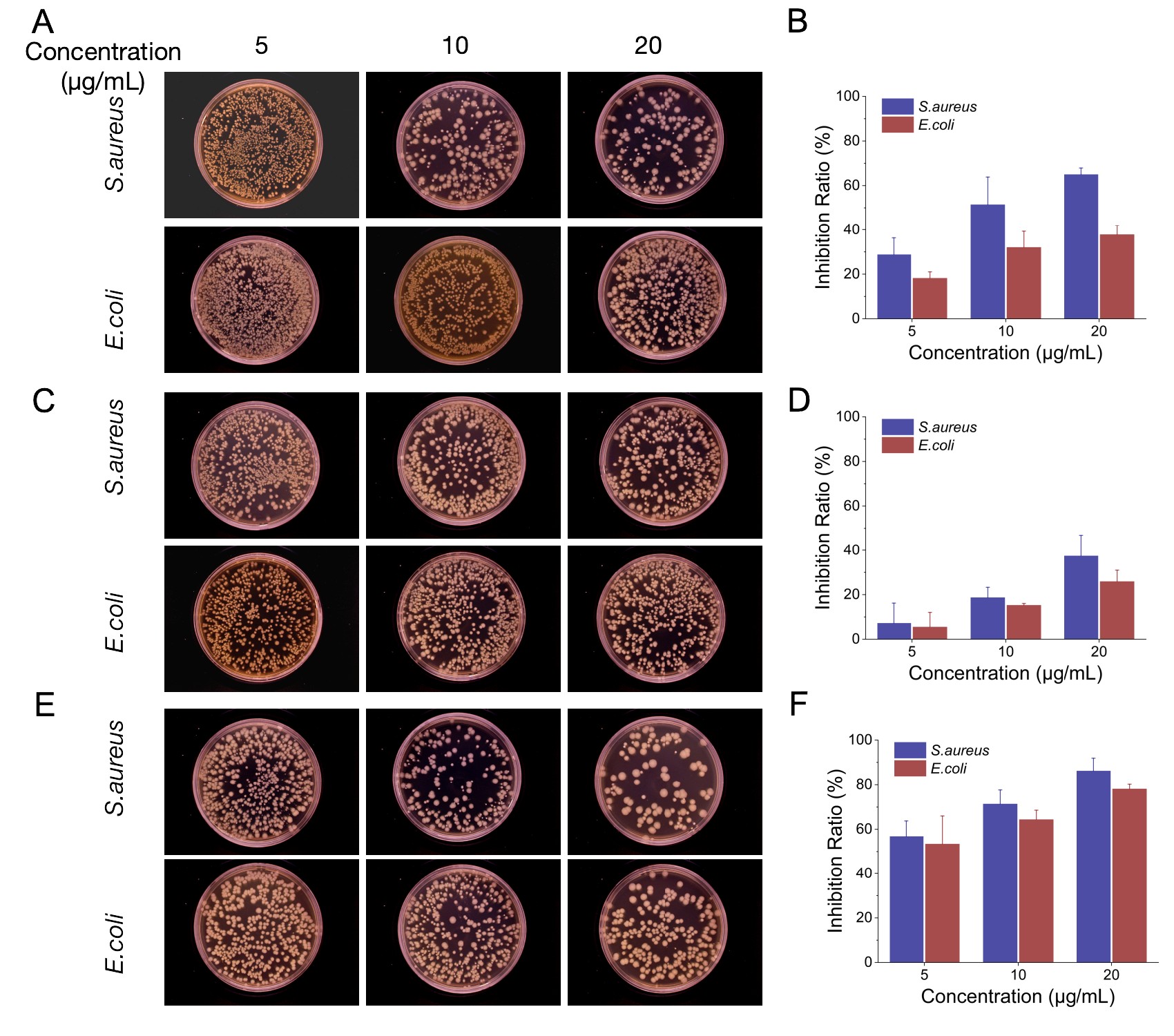


**Figure S5** Dose-dependent antibacterial activity of control formulations against *S. aureus* and *E. coli*. (A, B) Representative images of agar plates and the corresponding quantitative analysis of inhibition ratios for bacteria treated with various concentrations of various formulations: CuCl_2_. (C, D) Representative images of agar plates and the corresponding quantitative analysis of inhibition ratios for bacteria treated with various concentrations of free GS. (E, F) Representative images of agar plates and the corresponding quantitative analysis of inhibition ratios for bacteria treated with various concentrations of a physical mixture of GS & CuCl_2_.


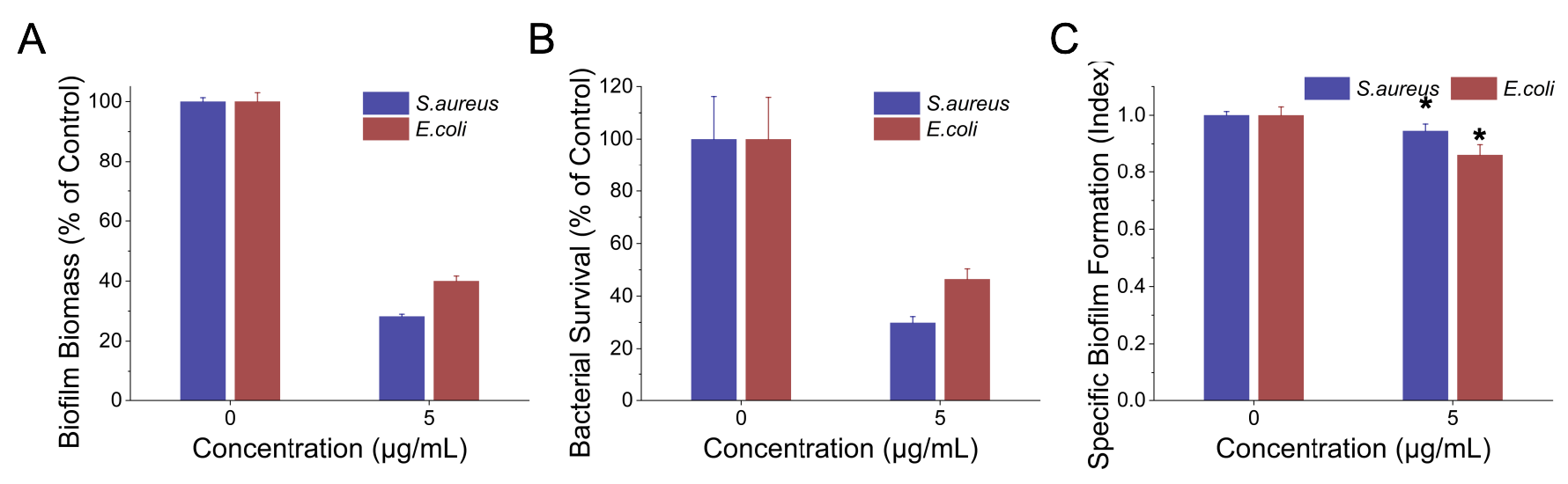


**Figure S6** Evaluation of specific anti-biofilm activity of GSR NPs normalized to bacterial survival. (A) Total biofilm biomass of *S. aureus* and *E. coli* treated with GSR NPs (5 µg/mL) expressed as a percentage of the control. (B) Planktonic bacterial survival under the same treatment conditions.

1. Specific Biofilm Formation (SBF) index, calculated as the ratio of biofilm biomass to bacterial survival (Biofilm/Growth), to distinguish specific anti-biofilm activity from bactericidal effects. Data are presented as mean ± SD (n=3). **P* < 0.05 compared to the Control group.
